# Dr. James Frederick William Ross

**Published:** 1912-01

**Authors:** 


Dr. James Frederick William Ross, Trinity, 1878, died of double pneumonia following an automobile accident, November 17, 1911, aged 55. He was prominent as a gynaecologist and surgeon.

